# Endophyte‐Derived Metabolites From the Bark of *Xylocarpus mekongensis*: Source of Antioxidant, Antimicrobial, and Antidiabetic Agents

**DOI:** 10.1155/tswj/6652670

**Published:** 2025-12-15

**Authors:** Sadia Airin, Rahul Dev Bairagi, Sharika Noshin, Raiyan Rahman Reon, Md. Sohanur Rahaman, Anike Chakrabarty, Amit Kumar Acharzo, Md. Amirul Islam

**Affiliations:** ^1^ Pharmacy Discipline, School of Life Sciences, Khulna University, Khulna, Bangladesh, ku.ac.bd; ^2^ Department of Medicine, Khulna Medical College Hospital, Khulna, Bangladesh

**Keywords:** *α*-Glucosidase, antimicrobial, antioxidant, endophytic, fungus, Sundarbans

## Abstract

Endophytic fungi have emerged as promising reservoirs of pharmacologically potent metabolites, garnering increasing scientific interest over the past three decades. Their ability to enhance host resilience against diverse biotic and abiotic stresses further underscores their biotechnological value. This study explores the hypothesis that endophytes from mangrove ecosystems, specifically the bark of *Xylocarpus mekongensis*, thrive under extreme conditions such as high salinity, humidity, temperature, and variable soil composition and may therefore produce unique bioactive compounds. The endophytic fungi were initially cultured in potato dextrose broth (PDB). The crude fungal extract was then obtained by solvent extraction, where the broth was first extracted with n‐hexane to remove nonpolar compounds, followed by extraction with ethyl acetate, which yielded the crude extract containing secondary metabolites. This procedure led to the successful isolation of three distinct culturable fungal endophytes, designated as X2, X4, and X7, and assessed for antimicrobial, antioxidant, and *α*‐glucosidase inhibitory properties through solvent fractionation. Among the isolates, X4 exhibited the most compelling pharmacological profile. Crude extracts of X4 demonstrated notable antioxidant activity in DPPH free radical scavenging activity (IC_50_: 94.179 *μ*g/mL), supported by high total phenolic content (TPC: 66.542 mg GAE/g), total flavonoid content (TFC: 173.770 mg QE/g), and total tannin content (TTC: 42.717 mg GAE/g), although still less potent than standard ascorbic acid (IC_50_: 15.987 *μ*g/mL). All crude and fractionated extracts exhibited measurable antibacterial activity, with X4 crude extract showing the strongest inhibition zones against *Escherichia coli* and *Bacillus subtilis* (21 mm). No antifungal effects were observed. Minimum inhibitory concentrations ranged from 31.5 to 250 *μ*g/mL. Additionally, the X4 isolate and its fractions displayed significant *α*‐glucosidase inhibition, with the crude extract showing the lowest IC_50_ (0.416 mg/mL), outperforming its ethyl acetate (0.824 mg/mL) and dichloromethane (1.032 mg/mL) fractions. These findings affirm that *X. mekongensis* bark harbors potent endophytic fungi capable of producing bioactive metabolites with strong therapeutic potential.

## 1. Introduction

The global burden of both noncommunicable and infectious diseases remains severe, with existing drugs for conditions like diabetes, bacterial infections, cancer, HIV/AIDS, hypertension, and malaria often limited by side effects and insufficient efficacy [1]. There is a pressing need to move beyond conventional “blockbuster” pharmaceutical R&D and turn back to nature, a historically reliable source, for innovative and effective drug discovery solutions [2]. Early humans likely discovered natural medicines through trial and error, driven by the urgent need to relieve disease symptoms. Long before written history, this knowledge was passed down orally across generations. Over time, numerous natural sources were identified for their healing properties and used to treat a wide range of health conditions [3–5]. Mangroves are vital for tropical marine ecosystems, rich in biodiversity and genetic resources. About 90% of marine species depend on them during part of their life cycle [6]. Mangrove plants and some endophytes are widely used in traditional medicine, and their extracts show activity against various pathogens. Few studies have identified the exact bioactive metabolites involved [7, 8]. An endophyte is an endosymbiont, often a bacterium or fungus, that lives within a plant for at least part of its life cycle without causing apparent disease. Endophytes are ubiquitous and have been found in all species of plants studied to date [9]. However, most of the endophyte/plant relationships are not well understood. Some endophytes may enhance host growth and nutrient acquisition and improve the plant′s ability to tolerate abiotic stresses, such as drought, and decrease biotic stresses by enhancing plant resistance to insects, pathogens, and herbivores [10, 11]. Most of the microbes colonizing the plants internally play key roles in the plant′s fitness and growth. A minor fraction of them may also cause diseases [12]. The overall interaction, however, is mutually beneficial. Thousands of microorganisms can be the inhabitants of a single plant, either in the form of epiphytes in the phyllo spheric region or as endophytes within tissues of leaves, roots, or stems. Endophytes display extensive diversity [13, 14]. Endophytic fungi produce bioactive metabolites with strong pharmaceutical potential [15]. Notable examples include guanacastepene, active against resistant bacteria [16]; Cytonic Acids A and B, which inhibit hCMV protease [17]; hinnuliquinone, a potent HIV‐1 protease inhibitor [18]; and antioxidant‐rich pestacin and isopestacin [19, 20]. L‐783,281 mimics insulin and activates neuroregenerative pathways [21], while Subglutinol A and B show immunosuppressive effects without cytotoxicity [22]. Paclitaxel (Taxol), a well‐known anticancer drug, also originates from endophytes [23]. Medicinal plants from extreme environments like the Sundarbans mangroves in the coastal regions of Bangladesh and India are rich in unique bioactive compounds due to their adaptation to salinity, flooding, and microbial pressure. As a biodiversity hotspot, the Sundarbans offers a valuable source of medicinal flora with strong potential for novel drug discovery.

Members of the *Xylocarpus* genus, including *Xylocarpus mekongensis*, are widely valued in traditional medicine. The bark of *X. mekongensis* is used for the treatment of malaria, diarrhea, inflammation, antinociceptive conditions, and oxidative stress, while its fruits are employed in managing elephantiasis and preventing breast swelling [24]. Despite these extensive ethnomedicinal applications and demonstrated pharmacological properties, such as anti‐inflammatory and antioxidant activity in its kernel root [25], the endophytic fungi associated with *X. mekongensis* remain largely unexplored. This gap highlights its potential as a promising source of novel bioactive compounds, since endophytes are known to produce metabolites with significant pharmacological activities. In mangrove ecosystems, endophytes from other plant hosts have been reported to yield diverse secondary metabolites with antimicrobial, antioxidant, anticancer, and enzyme inhibitory properties; for instance, *Trichoderma lentiforme* isolated from mangroves produces antimicrobial polyketides [26], while *Daldinia eschscholtzii* obtained from mangrove wood synthesizes metabolites with *α*‐glucosidase inhibitory and antibacterial activities [27, 28]. The study is aimed at assessing the antioxidant, antimicrobial, and *α*‐glucosidase inhibitory activities of the fungal endophytes, with a focus on identifying strains with strong therapeutic potential for developing novel drugs targeting oxidative stress, microbial infections, and metabolic disorders.

## 2. Material and Methods

### 2.1. Chemicals and Reagents

Seventy percent ethanol, chloramphenicol, potato dextrose agar (PDA) media, sodium hypochlorite, lactophenol, n‐hexane, dichloromethane (DCM), Ethyl acetate, Methanol, DPPH (2,2‐diphenyl‐1‐picrylhydrazyl), DMSO (dimethyl sulfoxide), ascorbic acid, Folin–Ciocalteu (FC) reagent, gallic acid, sodium carbonate (Na_2_CO_3_), 15% w/v sodium nitrate (NaNO_2_), 10% w/v aluminum chloride (AlCl_3_), 1 M sodium hydroxide (NaOH), quercetin, standard antibiotic discs (kanamycin 30 *μ*g/disc and fluconazole 50 *μ*g/disc), distilled water, alpha‐glucosidase enzyme from *Saccharomyces cerevisiae*, p‐nitrophenyl‐α‐d‐glucopyranoside (pNPG), KH_2_PO_4_, acarbose, and other chemicals, as well as solvents. Most chemicals are bought from Sigma Chemical Co. Ltd. in St. Louis, Missouri, United States. All reagents and solvents were of analytical grade.

### 2.2. Plant Collection

The plant *X. mekongensis* (family: Meliaceae) was collected from Koromjol of Sundarbans, Khulna, Bangladesh, on 16 February 2019, and adulteration was strictly forbidden during collection.

### 2.3. Isolation and Identification of Fungi

Plant parts were first thawed, washed with distilled water, and cut into small pieces. The samples were surface sterilized using ethanol and sodium hypochlorite, then rinsed and blotted dry. Sterilized tissues were placed on PDA plates containing chloramphenicol and incubated at room temperature for 1–3 weeks to allow fungal growth. Distinct fungal colonies were subcultured on fresh PDA to obtain pure isolates. For identification, fungi were cultured on PDA at 25°C for 5 days. Macroscopic characteristics (shape, size, color, and texture) were observed, and microscopic features (hyphae and conidia) were examined using the slide culture technique, stained with lactophenol cotton blue [29].

### 2.4. Extraction of Fungi

Endophytic fungi were first cultured in PDB and incubated at 28°C for 21 days on a rotary shaker at 150 rpm. After incubation, the fungal mycelia were aseptically separated from the broth using sterile cotton. The remaining broth was transferred to a separatory funnel and extracted with n‐hexane in a 2:1 ratio to remove nonpolar compounds. The upper hexane layer was separated, and the residual broth was further extracted with ethyl acetate to isolate secondary metabolites. The solvent layers were collected and evaporated using a rotary evaporator at 37°C to obtain crude fungal extracts. For further purification, solvent–solvent extraction (partitioning) was performed. A total of 150 mg of the methanolic extract obtained through cold extraction was dissolved in 100 mL of water and transferred into a separating funnel. Three immiscible organic solvents, n‐hexane, DCM, and ethyl acetate, were added (100 mL each) to the funnel based on increasing polarity. After each addition, the mixture was gently shaken, allowed to separate into layers, and the respective solvent layer was collected. Nonpolar compounds partitioned into the n‐hexane layer, moderately polar compounds into the DCM layer, and polar compounds into the ethyl acetate layer. This process was repeated to ensure effective separation of compounds according to their polarity [29, 30].

### 2.5. DPPH Free Radical Scavenging Activity

The antioxidant activity of the fungal extract was assessed using the DPPH free radical scavenging assay. Ten concentrations of each fungal extract and ascorbic acid (1, 2, 4, 8, 16, 32, 64, 128, 256, and 512 *μ*g/mL) were prepared in DMSO by the dilution technique, with ascorbic acid serving as the positive control. A 0.007886% (w/v) DPPH solution in methanol was prepared and homogenized using a sonicator. In a 96‐well microplate, 10 *μ*L of each concentration of sample and standard was added to 190 *μ*L of DPPH solution, maintaining a final DPPH concentration of 200 *μ*M. Each concentration was tested in triplicate, with 190 *μ*L of DPPH solution used as a blank. After mixing for 5 min, the plate was incubated at room temperature in the dark for 30 min, and absorbance was measured at 517 nm using a microplate reader. The IC_50_ value was determined from the % inhibition versus log concentration curve.

The % of inhibition was calculated as follows:

%inhibition=blank absorbance−sample absorbanceblank absorbance×100

### 2.6. Determination of Total Phenolic Content (TPC)

The TPC of the fungal extracts was quantitatively evaluated using the FC colorimetric method, with gallic acid employed as the analytical‐grade reference standard. The results were expressed in terms of milligrams of gallic acid equivalents per gram (mg GAE/g) of dried extract, calculated using a standard calibration curve derived from gallic acid solutions [31].

For the assay, 0.5 mL of each standard gallic acid solution (concentrations: 0.5, 0.4, 0.3, 0.2, and 0.1 mg/mL) and 0.5 mL of the fungal extract were transferred into separate test tubes. Subsequently, 5 mL of diluted FC reagent (1:10 dilution) and 4 mL of 7% Na_2_CO_3_ solution were added to each tube. The mixtures were vortexed thoroughly for 15 s to ensure homogeneity. Distilled water was added to bring the total volume of each reaction mixture to 10 mL. The test tubes were then incubated at 40°C for 30 min to facilitate color development through complexation of phenolic compounds with the FC reagent. Following incubation, the absorbance of each sample was measured at 765 nm using a UV‐visible spectrophotometer. Measurements were performed in duplicate for each concentration, and the mean absorbance was used for data analysis to ensure precision. A reagent blank was prepared under identical conditions, omitting both the sample and standard, to calibrate the spectrophotometer baseline.

### 2.7. Determination of Total Flavonoid Content (TFC)

The TFC of the sample extract was determined spectrophotometrically, using quercetin as the calibration standard [31, 32]. Results were expressed in milligrams of quercetin equivalents (mg QE) per gram of dried sample.

Initially, 1 mL of quercetin standard solutions at varying concentrations (0.5, 0.4, 0.3, 0.2, and 0.1 mg/mL) and 1 mL of the test extract were dispensed into individual test tubes. To each tube, 4 mL of distilled water and 0.3 mL of 5% (w/v) NaNO_2_ solution were added. The reaction mixtures were allowed to stand for 5 min at room temperature. Following this, 0.3 mL of 10% (w/v) AlCl_3_ solution and 2 mL of 1 M NaOH were added sequentially. The mixtures were then diluted with distilled water to a final volume of 10 mL per test tube. The solutions were incubated at ambient temperature for 15 min to allow for complex formation. After incubation, the absorbance of each reaction mixture was measured at 510 nm using a UV‐visible spectrophotometer, with an appropriate blank used to zero the instrument. Each measurement was performed in triplicate to ensure analytical reliability, and the mean absorbance values were applied to a standard calibration curve of quercetin to determine the flavonoid content in the samples.

### 2.8. Determination of Total Tannin Content (TTC)

The TTC was determined using the FC method [33], where 0.1 mL of standard gallic acid solutions of different concentrations (0.5, 0.4, 0.3, 0.2, and 0.1 mg/mL) and the extract sample were taken separately in test tubes. To each tube, 7.5 mL of distilled water was added, followed by 0.5 mL of FC reagent and 1 mL of 35% Na_2_CO_3_ solution, and the volume was made up to 10 mL with distilled water. The mixtures were vortexed for 15 s and incubated at room temperature for 30 min. Absorbance was then measured at 725 nm using a spectrophotometer, while a blank was prepared by following the same procedure without the addition of gallic acid or sample.

### 2.9. Antimicrobial Assay

The antimicrobial efficacy of the fungal crude extracts (X2, X4, and X7) and their solvent‐partitioned fractions (n‐hexane, DCM, and ethyl acetate) was meticulously evaluated through both disc diffusion and minimum inhibitory concentration (MIC) assays [34–36]. Initially, PDA was prepared by decocting unpeeled potatoes, incorporating dextrose and agar, followed by sterilization via autoclaving at 121°C under 15 Ibs/sq.in. pressure for 15 min. All culture media, glassware, and instruments were aseptically maintained within a laminar airflow cabinet preirradiated with ultraviolet light to preclude microbial contamination. The bacterial strains employed included *Staphylococcus aureus* and *Bacillus subtilis* (Gram positive) and *Escherichia coli* and *Salmonella enterica* (Gram negative), alongside the fungal strain *Candida albicans*, all of which were obtained from the microbiology laboratory and subcultured to log phase on PDA slants.

The fungal extracts and fractions were solubilized in ethanol to obtain precise concentrations of 250 *μ*g/100 *μ*L and 500 *μ*g/100 *μ*L. These solutions were then meticulously applied to sterile 5‐mm diameter filter paper discs, which were carefully placed onto agar plates preinoculated with test organisms. Positive controls comprised ciprofloxacin (30 *μ*g/disc) and fluconazole (50 *μ*g/disc), while negative controls consisted of ethanol‐impregnated blank discs. The inoculated plates were incubated at 37°C for 16–18 h (bacterial cultures) and 48–72 h (fungal cultures). Antimicrobial potency was quantified by measuring the diameter of growth inhibition zones surrounding the discs.

Subsequently, the MIC of extracts demonstrating inhibitory activity was assessed using the broth microdilution technique in a 96‐well format. Serial twofold dilutions of extract stock solutions were prepared in potato dextrose broth containing DMSO. Each well received 100 *μ*L of diluted extract and 100 *μ*L of microbial suspension (~5 × 10^5^ CFU/mL). Gentamicin was employed as the reference antibiotic. The microplates were incubated at 37°C for 18–24 h, and bacterial proliferation was visually assessed based on turbidity. The MIC was determined as the lowest concentration of the extract that completely abrogated visible microbial growth, signifying its bacteriostatic or bactericidal potential. All antimicrobial assays were performed in triplicate to ensure the reliability and reproducibility of the results. The inhibition zone diameters and MIC values were expressed as mean ± standard deviation (SD). Statistical analysis was carried out to minimize experimental error and validate the consistency of the observed antimicrobial activity among replicates, thereby strengthening the significance of the findings.

### 2.10. Alpha‐Glucosidase Enzyme Inhibitory Activity

The *α*‐glucosidase inhibitory potential of the extracts was assessed utilizing a modified colorimetric protocol in a 96‐well microtiter plate format. Each reaction mixture consisted of 50 *μ*L of potassium phosphate buffer (PBS, pH 6.8), 10 *μ*L of *α*‐glucosidase enzyme solution (1 unit/mL), and 20 *μ*L of the test extract at graded concentrations (0.1–0.5 mg/mL), predissolved in methanol. These mixtures were preincubated at 37°C for 15 min to facilitate optimal enzyme–substrate interaction. Subsequently, 20 *μ*L of the substrate solution, *p*‐nitrophenyl‐*α*‐d‐glucopyranoside (pNPG), was introduced to initiate the enzymatic reaction, followed by a second incubation at 37°C for an additional 15 min. To quench enzymatic activity and terminate the reaction, 50 *μ*L of Na_2_CO_3_ solution was added to each well. The extent of enzymatic hydrolysis was quantified by measuring the absorbance at 405 nm using a microplate spectrophotometer. As a positive control, 20 *μ*L of standard acarbose solution was employed in place of the test extract, while 20 *μ*L of methanol served as the negative (blank) control. The assay enabled the evaluation of inhibitory efficacy by comparing absorbance values between test samples, standards, and controls [37].

## 3. Results

### 3.1. DPPH Free Radical Scavenging Activity

**Figure 1:**
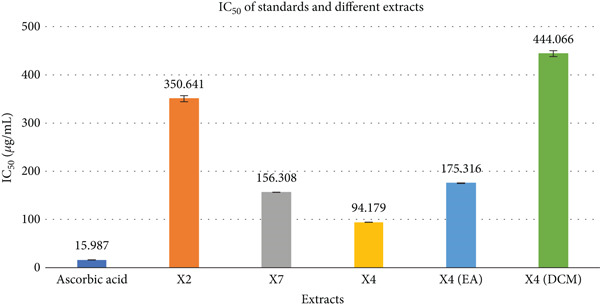
Comparison of DPPH scavenging assay by ascorbic acid and various samples.

The antioxidant activity of various fungal extracts was quantitatively evaluated via their dose‐dependent DPPH radical scavenging capacity. Among them, the methanolic extract of strain X4 exhibited notable inhibition with an IC_50_ of 94.12 ± 0.19 *μ*g/mL, whereas the reference compound, ascorbic acid, demonstrated substantially higher potency, IC_50_ = 15.98 ± 0.19 *μ*g/mL (Figure 1). In contrast, the X2 extract showed poor radical scavenging ability (IC_50_ = 350.64 ± 6.39 *μ*g/mL). The X4 fractions displayed variable activity—ethyl acetate fraction showed moderate inhibition (IC_50_ = 175.32 ± 0.75 *μ*g/mL), while the DCM fraction exhibited weak activity (IC_50_ = 444.06 ± 6.29 *μ*g/mL). The X7 extract also demonstrated moderate scavenging potential, with an IC_50_ of 156.31 ± 0.249 *μ*g/mL. Percent inhibition versus log concentration for ascorbic acid shows a linear relationship described by the equation *y* = 37.529*x* + 4.8256 with a coefficient of determination *R* ^2^ = 0.9838. Full data are summarized in Figure 1.

### 3.2. TPC

**Figure 2:**
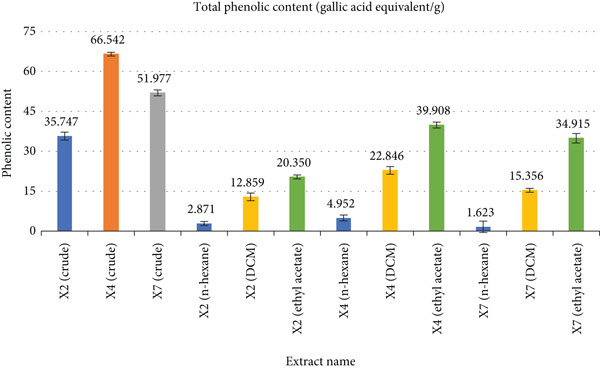
Comparison of total phenolic content determined from various extracts.

The TPC of fungal extracts was determined at 1 mg/mL (765 nm) using a gallic acid standard calibration curve (Figure 2). Among the crude extracts, X4 exhibited the highest TPC (66.542 ± 0.721 mg GAE/g), followed by X7 (51.977 ± 1.101) and X2 (35.747 ± 1.500). Fraction‐wise, TPC values for X2 were 2.871 ± 0.721 (n‐hexane), 12.859 ± 1.442 (DCM), and 20.350 ± 0.721 (ethyl acetate); for X4: 4.952 ± 1.101 (n‐hexane), 22.846 ± 1.442 (DCM), and 39.908 ± 1.101 (ethyl acetate); and for X7: 1.623 ± 2.162 (n‐hexane), 15.356 ± 0.721 (DCM), and 34.915 ± 1.814 (ethyl acetate). The TPC of different fungal extracts was determined using a gallic acid standard calibration curve, showing a linear relationship described by the equation *y* = 0.801*x* + 0.0167 with a coefficient of determination *R* ^2^ = 0.993. Full data are summarized in Figure 2.

### 3.3. TFC

**Figure 3:**
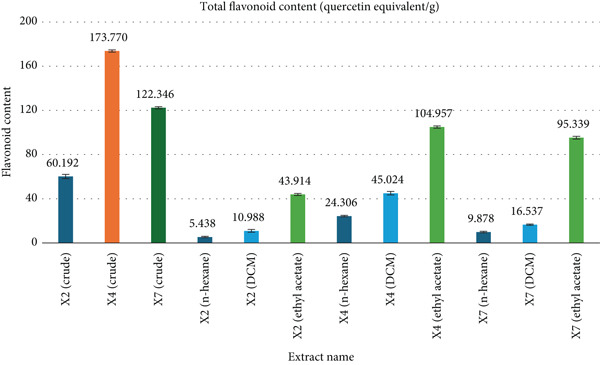
Comparison of total flavonoid content determined from various extracts.

The TFC of fungal extracts was measured at 510 nm (1 mg/mL concentration) using a quercetin standard calibration curve (Figure 3). Among the crude extracts, X4 showed the highest TFC (173.770 ± 0.979 mg QE/g), followed by X7 (122.346 ± 0.979) and X2 (60.192 ± 1.958). Fraction‐wise, X2 exhibited TFC values of 5.438 ± 0.641 (n‐hexane), 10.988 ± 1.282 (DCM), and 43.914 ± 0.979 (ethyl acetate). X4 fractions showed 24.306 ± 0.641 (n‐hexane), 45.024 ± 1.613 (DCM), and 104.957 ± 0.979 (ethyl acetate). X7 fractions recorded 9.878 ± 0.641 (n‐hexane), 16.537 ± 0.641 (DCM), and 95.339 ± 1.282 mg QE/g (ethyl acetate). The TFC of different extracts was determined using a quercetin standard calibration curve, showing a linear relationship described by the equation *y* = 0.901*x* + 0.0741 with a coefficient of determination *R* ^2^ = 0.9907. Full data are summarized in Figure 3.

### 3.4. TTC

**Figure 4:**
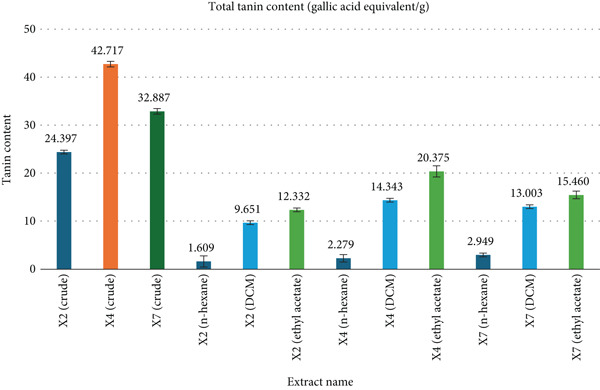
Comparison of total tannin content determined from various extracts.

The TTC of fungal extracts was evaluated at 725 nm using a 1 mg/mL concentration. Gallic acid was used as the standard for calibration (Figure 4), and TTC values were expressed in mg GAE/g along with the standard error of the mean (SEM). Among the crude extracts, X4 exhibited the highest TTC (42.717 ± 0.591 mg GAE/g), followed by X7 (32.887 ± 0.591) and X2 (24.397 ± 0.387). In the X2 fractions, TTC values were 1.609 ± 1.161 (n‐hexane), 9.651 ± 0.387 (DCM), and 12.332 ± 0.387 (ethyl acetate). X4 fractions showed 2.279 ± 0.774 (n‐hexane), 14.343 ± 0.387 (DCM), and 20.375 ± 1.161 (ethyl acetate), while X7 fractions recorded 2.949 ± 0.387 (n‐hexane), 13.003 ± 0.387 (DCM), and 15.460 ± 0.806 mg GAE/g (ethyl acetate). TTC determination of different extracts using gallic acid standard calibration curve showed a linear relationship described by the equation *y* = 1.492*x* + 0.0556, *R* ^2^ = 0.9904. Full data are summarized in Figure 4.

### 3.5. Antimicrobial Assay

Among the tested extracts, X4 ethyl acetate showed the strongest antibacterial activity (ZOIs 17.1–19.1 mm; MIC 31.25–62.5 *μ*g/mL), followed by X4 DCM (moderate activity) and X4 n‐hexane (weak or inactive). X7 crude and fractions exhibited moderate to low activity, while X2 crude showed low activity. Gentamicin had the lowest MICs (0.78–3.13 *μ*g/mL). Full data are in Tables 1 and 2.

**Table 1:** In vitro antibacterial activity of different extracts (diameter of zone of inhibition [ZOI] in millimeter).

**Name of extract**	***Staphylococcus aureus***			***Bacillus subtilis***			***Escherichia coli***			***Salmonella enterica***		
	**ZOI 1**	**ZOI 2**	**A** **v** **e** **r** **a** **g** **e** ± **S** **E** **M**	**ZOI 1**	**ZOI 2**	**A** **v** **e** **r** **a** **g** **e** ± **S** **E** **M**	**ZOI 1**	**ZOI 2**	**A** **v** **e** **r** **a** **g** **e** ± **S** **E** **M**	**ZOI 1**	**ZOI 2**	**A** **v** **e** **r** **a** **g** **e** ± **S** **E** **M**
Standard (ciprofloxacin 30 *μ*g/disc)	25	25.5	25.25 ± 0.25	28	28.4	28.2 ± 0.2	30	29.7	29.85 ± 0.15	29	29.5	29.25 ± 0.25
Control (blank)	0		0	0		0	0		0	0	0	0
X2 crude extract (250 *μ*g/disc)	8	8.3	8.15 ± 0.15	8	7.8	7.9 ± 0.1	8	8.5	8.25 ± 0.25	9	8.7	8.85 ± 0.15
X2 crude extract (500 *μ*g/disc)	9	8.8	8.9 ± 0.1	9	9.3	9.15 ± 0.15	8.5	8.7	8.6 ± 0.10	9	8.8	8.9 ± 0.10
X2 ethyl acetate fraction (250 *μ*g/disc)	8	8.4	8.2 ± 0.2	8	7.7	7.85 ± 0.15	9	9.2	9.1 ± 0.10	7	7.5	7.25 ± 0.25
X2 ethyl acetate fraction (500 *μ*g/disc)	8	8.3	8.15 ± 0.15	9	9.2	9.1 ± 0.1	8.5	8.8	8.65 ± 0.15	8	8.3	8.15 ± 0.15
X2 DCM fraction (250 *μ*g/disc)	7	7.4	7.2 ± 0.2	8	7.7	7.85 ± 0.15	8	8.2	8.1 ± 0.10	8	7.6	7.8 ± 0.20
X2 DCM fraction (500 *μ*g/disc)	7.5	7.7	7.6 ± 0.1	9	8.9	8.95 ± 0.05	8	8.3	8.15 ± 0.15	7	7.7	7.35 ± 0.35
X4 crude extract (250 *μ*g/disc)	17	17.3	17.15 ± 0.15	13	13.5	13.25 ± 0.25	17	16.8	16.9 ± 0.10	16	15.7	15.85 ± 0.15
X4 crude extract (500 *μ*g/disc)	19	18.7	18.85 ± 0.15	21	21.2	21.1 ± 0.1	21	20.7	20.85 ± 0.15	19	19.3	19.15 ± 0.15
X4 ethyl acetate fraction (250 *μ*g/disc)	15	15.3	15.15 ± 0.15	11	11.4	11.2 ± 0.20	15	14.8	14.9 ± 0.10	13	12.8	12.9 ± 0.10

**Table 2:** In vitro antibacterial activity of different extracts (diameter of zone of inhibition [ZOI] in millimeter).

**Name of extract**	***Staphylococcus aureus***			***Bacillus subtilis***			***Escherichia coli***			***Salmonella enterica***		
	**ZOI 1**	**ZOI 2**	**A** **v** **e** **r** **a** **g** **e** ± **S** **E** **M**	**ZOI 1**	**ZOI 2**	**A** **v** **e** **r** **a** **g** **e** ± **S** **E** **M**	**ZOI 1**	**ZOI 2**	**A** **v** **e** **r** **a** **g** **e** ± **S** **E** **M**	**ZOI 1**	**ZOI 2**	**A** **v** **e** **r** **a** **g** **e** ± **S** **E** **M**
X4 ethyl acetate fraction (500 *μ*g/disc)	17	17.2	17.1 ± 0.10	19	18.7	18.85 ± 0.15	19	19.2	19.1 ± 0.10	19	18.7	18.85 ± 0.15
X4 DCM fraction (250 *μ*g/disc)	12	12.3	12.15 ± 0.15	9	8.9	8.95 ± 0.05	14	13.8	13.9 ± 0.10	11	11.6	11.3 ± 0.30
X4 DCM fraction (500 *μ*g/disc)	17	16.9	16.95 ± 0.05	16	16.2	16.1 ± 0.10	18	17.7	17.85 ± 0.15	18	18.4	18.2 ± 0.20
X4 n‐hexane fraction (250 *μ*g/disc)	0	0	0	6	6.3	6.15 ± 0.15	6	6.2	6.1 ± 0.10	0	0	0
X4 n‐hexane fraction (500 *μ*g/disc)	0	0	0	8	8.4	8.2 ± 0.20	6.5	6.2	6.35 ± 0.15	0	0	0
X7 crude extract (250 *μ*g/disc)	9	9.2	9.1 ± 0.10	7.5	7.8	7.65 ± 0.15	10	9.7	9.85 ± 0.15	9	8.8	8.9 ± 0.10
X7 crude extract (500 *μ*g/disc)	13	13.3	13.15 ± 0.15	16	15.7	15.85 ± 0.15	12	12.2	12.1 ± 0.10	13	13.3	13.15 ± 0.15
X7 ethyl acetate fraction (250 *μ*g/disc)	9	9.5	9.25 ± 0.25	6	6.5	6.25 ± 0.25	8.5	8.7	8.6 ± 0.10	7	7.2	7.1 ± 0.10
X7 ethyl acetate fraction (500 *μ*g/disc)	11	10.8	10.9 ± 0.10	13	13.2	13.1 ± 0.10	11.5	11.4	11.45 ± 0.05	10	9.7	9.85 ± 0.15
X7 DCM fraction (250 *μ*g/disc)	7	7.3	7.15 ± 0.15	6	6.4	6.2 ± 0.20	6	6.3	6.15 ± 0.15	6.5	6.7	6.6 ± 0.10
X7 DCM fraction (500 *μ*g/disc)	11	11.2	11.1 ± 0.10	11	10.8	10.9 ± 0.10	9	9.3	9.15 ± 0.15	9	9.2	9.1 ± 0.10

### 3.6. *α*‐Glucosidase Inhibitory Activity

**Figure 5:**
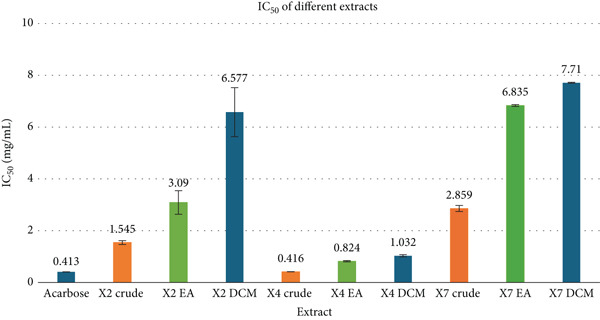
Comparison of *α*‐glucosidase inhibitory activity of different extracts.

The standard drug acarbose exhibited the strongest *α*‐glucosidase inhibition with an IC_50_ of 0.413 ± 0.004 mg/mL. Among the extracts, the X4 crude extract showed comparable potency (0.416 ± 0.004 mg/mL), followed by X4 EA (0.824 ± 0.023) and X4 DCM (1.032 ± 0.042) fractions. In contrast, X2 crude and its fractions were less active, with IC_50_s of 1.545 ± 0.076 (crude), 3.09 ± 0.457 (EA), and 6.577 ± 0.947 (DCM). The X7 extract and fractions demonstrated the weakest inhibition, with IC_50_s of 2.859 ± 0.116 (crude), 6.835 ± 0.036 (EA), and 7.71 ± 0.017 mg/mL (DCM). Full data are summarized in Figure 5.

## 4. Discussion

Mangrove ecosystems host diverse endophytic fungi adapted to harsh saline conditions, making them rich sources of bioactive compounds. This study investigates endophytic fungi from the bark of *X. mekongensis*, highlighting their antioxidant, antibacterial, and *α*‐glucosidase inhibitory activities with potential against oxidative stress, infections, and Type 2 diabetes. Endophytic fungi from *X. mekongensis*, particularly isolate X4, showed strong antioxidant effects linked to phenolics, flavonoids, and tannins, alongside notable antibacterial activity against *E. coli* and *B. subtilis* (zones up to 21 mm) but no antifungal effect. X4 and its ethyl acetate fraction also displayed potent *α*‐glucosidase inhibition, comparable to acarbose, likely due to flavonoids such as luteolin and kaempferol, highlighting its potential for developing safer, natural therapies against oxidative stress, infections, and Type 2 diabetes [38, 39].

The comparative evaluation of antioxidant activity among different endophytic fungi highlights both shared and unique pharmacological potentials. The cultivated fruiting bodies of *Xylaria* sp. (YX‐28) from *Ginkgo biloba* showed strong antioxidant activity in DPPH and *β*‐carotene–linoleic acid systems, with the methanol extract containing the highest phenolic (54.51 ± 1.05 mg GAE/g DW) and flavonoid (86.76 ± 0.58 mg RE/g DW) levels, whereas the hexane extract had the lowest (9.71 ± 0.57 mg GAE/g and 10.14 ± 0.76 mg RE/g) [40]. Similarly, endophytes from *Dillenia indica* demonstrated potent antioxidant capacity, where over 60% of isolates showed 50%–90% activity [41]; the *Fomitopsis meliae* extract exhibited the highest inhibition (91.5%, IC_50_ = 88.27 *μ*g/mL), and *Chaetomium globosum* displayed 89.88% inhibition (IC_50_ = 74.44 *μ*g/mL) with TPC of 37.4 mg GAE/g and TFC of 31.0 mg QE/g, alongside GC‐MS‐identified phenolic metabolites such as squalene and octadecadienoic acid. By contrast, endophytic fungi isolated from *X. mekongensis* bark yielded three isolates, of which X4 was most active, exhibiting DPPH scavenging with IC_50_ = 94.179 *μ*g/mL, supported by comparatively higher TPC (66.542 mg GAE/g), TFC (173.770 mg QE/g), and TTC (42.717 mg GAE/g), though less potent than ascorbic acid (IC_50_ = 15.987 *μ*g/mL). In addition, X4 showed antibacterial activity (21 mm zone against *E. coli* and *B. subtilis*) and notable *α*‐glucosidase inhibition (IC_50_ = 0.416 mg/mL). Collectively, while *Xylaria* sp. and *D. indica* endophytes primarily underline antioxidant efficacy linked to phenolic content, the *X. mekongensis* isolate X4 displayed not only comparable antioxidant potential but also broader pharmacological activities, including antibacterial and enzyme inhibition, highlighting its unique therapeutic relevance [40–44]. Endophytic fungi show strong antidiabetic potential, with *X. mekongensis* isolate X4 exhibiting potent in vitro *α*‐glucosidase inhibition (IC_50_ = 0.416 mg/mL), while *Schizophyllum commune* (Sch1) from *Aloe vera* not only showed > 90% in vitro inhibition but also demonstrated significant in vivo antidiabetic effects in streptozotocin‐induced rats, making Sch1 the more comprehensive candidate [45]. Natural antioxidants are significant because they offer therapeutic potential as antioxidant drugs, providing protection against oxidative stress related diseases such as cancer, diabetes, cardiovascular, and neurodegenerative disorders. For example, flavonoids and phenolic compounds from endophytic fungi demonstrate strong radical scavenging activity, positioning them as safer and more sustainable alternatives to synthetic antioxidant drugs like BHA and BHT [46].

This study powerfully highlights the immense biopharmaceutical promise harbored within endophytic fungi isolated from mangrove species, particularly *X. mekongensis*. Among the isolates, the X4 extract stands out for its potent antioxidant, antibacterial, and antidiabetic properties, positioning it as a strong candidate for natural product‐based drug discovery. The extract′s robust performance across diverse bioassays establishes a compelling basis for advanced pharmacological investigations, including the purification and structural elucidation of active constituents, mechanistic studies, and in vivo efficacy evaluations. Future research must prioritize the isolation and characterization of individual bioactive compounds, as well as the exploration of potential synergistic interactions that could amplify therapeutic outcomes. Furthermore, in‐depth preclinical and clinical validations are essential to assess safety profiles and therapeutic relevance. Equally critical is the investigation of biosynthetic gene clusters within these fungal strains to facilitate scalable production through biotechnological means. The broader significance of this work lies in its dual contribution to sustainable drug development and the strategic exploitation of mangrove endophytes, an underexplored microbial niche with remarkable chemical diversity. At a time when antimicrobial resistance and chronic diseases such as diabetes pose escalating global health threats, this study reinforces the untapped potential of marine endophytes to serve as transformative resources in the development of next‐generation therapeutics.

## 5. Conclusion

This study highlights the promising therapeutic potential of endophytic fungi derived from *X. mekongensis*, particularly the X4 isolate, which demonstrated strong antioxidant, antimicrobial, and *α*‐glucosidase inhibitory activities. These findings suggest that mangrove‐associated endophytes may serve as valuable natural sources for future drug development initiatives targeting oxidative stress, infections, and metabolic disorders.

**Nomenclature**
DCM: dichloromethane
DPPH: 2,2‐diphenyl‐1‐picrylhydrazyl
TFC: total flavonoid content
TPC: total phenolic content
GAE: gallic acid equivalent
TTC: total tannin content
EA: ethyl acetate
MIC: minimum inhibitory concentration
PDA: potato dextrose agar
QE: quercetin equivalent
DMSO: dimethyl sulfoxide
FC: Folin–Ciocalteu
HIV: human immunodeficiency virus
UV: ultraviolet
CFU: colony‐forming unit
PBS: phosphate‐buffered saline
PDB: potato dextrose broth
SEM: standard error of the mean
ZOI: zone of inhibition

## Disclosure

All authors have read and approved the final manuscript and agree to be accountable for its contents.

## Conflicts of Interest

The authors declare no conflicts of interest.

## Author Contributions

Conceptualization: S.A., R.D.B., and M.A.I.; methodology: S.A., S.N., and M.S.R.; investigation: S.A., S.N., and A.K.A.; data curation and formal analysis: S.A., M.S.R., R.D.B., and M.S.R.; resources: R.R.R., A.C., and A.K.A.; visualization: S.A., S.N., and M.A.I.; writing—original draft: S.A., R.R.R., A.C., and R.D.B.; writing—review and editing: A.K.A. and M.A.I.; supervision: M.A.I.; project administration: M.A.I.

## Funding

No funding was received for this manuscript.

## Data Availability

The research data used to support the findings of this study are included within the article.
